# Laplace-domain diffuse optical measurement

**DOI:** 10.1038/s41598-018-30353-5

**Published:** 2018-08-14

**Authors:** Ali Hasnain, Kalpesh Mehta, Xiaowei Zhou, Hongsheng Li, Nanguang Chen

**Affiliations:** 1 Department of Biomedical Engineering, National University of Singapore, Singapore, 117576 Singapore; 2 Department of Radiology, Cancer Center, Guangzhou Medical University, Guangzhou, China

## Abstract

Time-domain diffuse optical measurement systems determine depth-resolved absorption changes by using the time of flight distribution of the detected photons. It is well known that certain feature data, such as the Laplace transform of the temporal point spread function, is sufficient for image reconstruction and diffuse optical sensing. Conventional time-domain systems require the acquisition of full temporal profiles of diffusive photons and then numerically compute the feature dataset, for example, Laplace transformed intensities for imaging applications. We have proposed a novel method for directly obtaining the Laplace transform data. Our approach can significantly improve the data acquisition speed for time-domain diffuse optical imaging. We also demonstrated that the use of negative Laplace parameters can provide enhanced sensitivity to perturbations located in deep regions.

## Introduction

A diffuse optical measurement system uses a near-infrared (NIR) spectral window where the absorption coefficients of water and hemoglobin are relatively small and therefore enjoy many advantages in terms of imaging depth, sensitivity and ability to provide functional information^1–3^. Continuous-wave domain (CW), frequency domain (FD) and time-domain (TD) are three different approaches to diffusive optical measurement. The CW domain method has the advantages of being low cost, fast, and portable but measures the combined contributions of photons diffusing through both superficial and deep tissues. The systemic physiological changes such as heart-beat and blood pressure may contaminate the desired signal from the deep layers; as they are associated with early arriving photons with much stronger intensity than the late-arriving photons from the deep regions. This fact limits the sensitivity for detecting the signal from the deep layers^4–6^. To improve the depth sensitivity, a CW domain system usually resorts to multi-distance source-detector pairs. FD systems require RF amplitude modulation of the laser source and measure the change in the amplitude and phase of the diffusive photon waves after passing through the sample. The modulation frequency ranges from 100–1000 MHz. Conventional TD systems use an ultrashort pulse to illuminate the tissue sample; the pulse spreads in time after passing through the sample and a histogram of the time of flight of photons, called temporal profile spread function (TPSF), is recorded. The optical properties are retrieved by analyzing the TPSF’s. Among all the three implementations, the time-domain technique provides the richest information and the best reconstruction accuracy^7,8^.

In conventional time-domain systems, an ultrashort pulsed laser is used to illuminate the tissue sample and the signal from the sample is detected using either a streak camera or a time correlated single photon counter (TCSPC). They have several limitations such as small dynamic range, temporal nonlinearity in the case of streak camera based detection system and slower data acquisition time and sensitivity to ambient environment in case of the TCSPC based system^9^. They are usually bulky, slow, expensive, complicated and less scalable to increase the number of source detector pairs.

Spread spectrum techniques have been used in communication systems for secure and high fidelity communication with many advantages such as low error rate, interference rejection, and selective addressing capability. In the spread spectrum method, a communication signal (electrical, electromagnetic or acoustic) with a particular bandwidth is deliberately spread in the frequency domain to transform it into a wide-band signal. These techniques require the use of pseudorandom number sequences (PRBS) to determine and control the spreading behavior of the signal within the bandwidth. A PRBS has a weak cross correlation with other sequences and a very desirable auto-correlation function, which is analogous to a delta function^10^. Therefore, the desired signal picked up by a receiver can be well distinguished from the environmental noises, interferences, and other different PRBS patterns. It is also straightforward to time the same sequence of PRBS arriving from multiple paths. This is the fundamental principle used in our design of time-resolved diffuse optical measurement system as it allows photons detected at different time delays to be resolved with sub-nanosecond time resolution. This spread spectrum technique has been employed to build compact and low cost time-resolved diffuse optical tomography systems with higher signal-to-noise ratio and faster data acquisition^11–15^.

The full time spectrum carries some redundant information from diffusive photons^16^. Many computational methods have been developed to extract featured data sets from the temporal profiles of diffusive photons and to use them for image reconstruction or spectroscopy studies. Such derived transforms include Laplace transform, Fourier transform, and Mellin tranform^17^. Practices have shown that high-order Mellin transforms are sensitive to noise^18^ and the periodic nature of Fourier transform usually induces strong oscillations^19^. Nonetheless, both the Laplace and Fourier transform have been the preferred methods for analyzing time-resolved data^20^. In the case of time-domain fluorescence diffuse optical tomography, these transformed data have simple and direct connections to the lifetime of fluorescence emission^21^.

In an implicit way, Laplace transform with positive and negative Laplace frequencies contain information about early arriving and late-arriving photons respectively. Laplace transform with positive Laplace frequency provides exponential weighting such that it emphasizes on early arriving photons, while Laplace transform with negative frequency weights more on late-arriving photons. In time-domain fluorescence diffuse optical tomography, these facts are used to selectively reconstruct using the early arriving photons^21–23^.

Acquisition of the full spectrum time domain data leads to unnecessarily extended measurement time while only Laplace transformed TPSF’s at several Laplace frequencies are needed for reconstructing optical properties and their spatial distributions. To further improve the data acquisition speed of our spread spectrum approach, we have developed a novel Laplace-domain diffuse optical measurement method. With the proposed method, it is possible to directly measure the Laplace transform of TPSF’s without the need of recording the complete temporal profiles. We believe that this method can significantly improve the imaging speed and achieve enhanced sensitivity for detecting deep region perturbation using late-arriving photons^3–6,20,24–27^.

## Methods

### System Design

Spread spectrum time-resolved optical measurement requires high-speed intensity modulation of the light source. There are usually two techniques for modulation: direct light modulation and direct current modulation of a laser source. Generally, direct light modulation can be achieved using an external acousto-optic modulator (AOM) or electro-optic modulator (EOM). AOMs are not suitable for fast modulation as their response speed is limited by the transit of sound wave across the beam diameter^28^. EOMs, usually based on interferometric modulation, can handle large power with a minimum optical loss but they are sensitive to the environment and have an inherent modulation instability which induces an unwanted bias drifting effect over a period of time. Very often they require a bias controller to dynamically monitor and adjust the bias, which makes the overall implementation complex.

Direct current modulation involves changing the input current of the laser diode to directly modulate the optical output. It is simple and cheap but at higher frequencies, the modulation response of edge-emitting diodes reduces as the injection signal becomes really small^29^. This problem can be removed by using a vertical-cavity surface-emitting laser (VCSEL) which has a good modulation response at higher frequencies but the optical power is usually low (a few mW). Direct electrical modulation of laser diodes is advantageous to using electro-optics modulators (EOM) such as Mach-Zehnder modulator (MZM)^30^, as they do not require any extra control. In our implementation, we chose to use the direct RF modulation technique to intensity modulate high speed 2.5 Gbps VCSELs from Thorlabs. With proper input impedance matching, a modulation depth of more than 80% was achieved.

**Figure 1:**
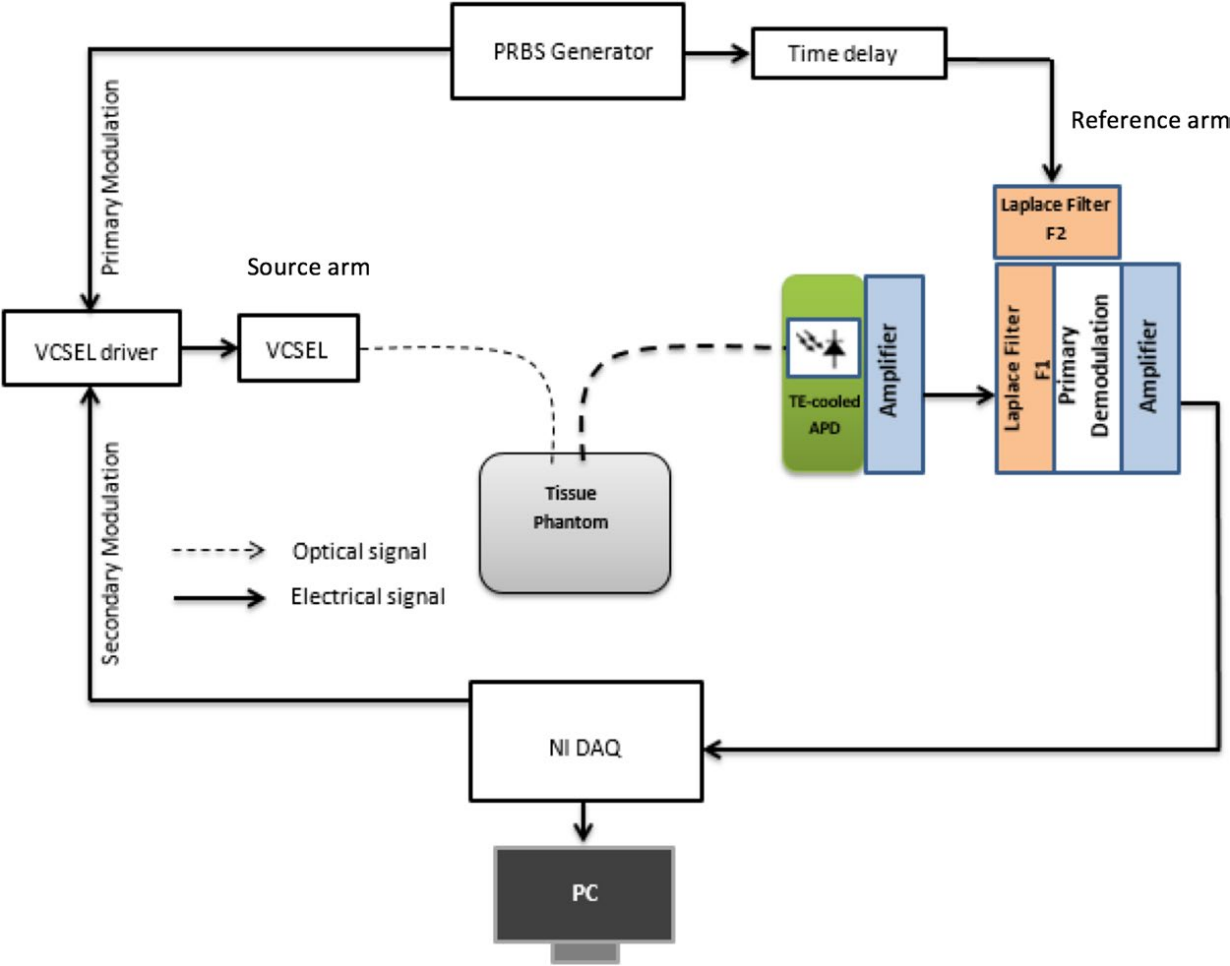
Schematic design of Laplace-domain DOS. The thick lines represent the electrical signal and the thin lines represent the optical signal.

The overall architecture of the system is illustrated in Fig. 1. The light source is a 2 mW 780 nm VCSEL which can be directly modulated up to 2.5 gigabit per second (Gbps). The modulation signal fed into the VCSEL is generated by a driver circuit, which is an analog mulitplier (ADL5391, Analog Devices) and mixes a primary modulation signal from a PRBS generator^3^ and a secondary modulation signal from an NI data acquisition (DAQ) device (USB6343, National Instruments). The primary PRBS modulation signal is generated at 2.5 Gbps, while the secondary modulation signal is a 20 kHz square wave. A high-speed avalanche photodiode (APD), which is thermal electrically cooled, is used to detect the diffusive photons that propagate through the sample under investigation. The photoelectrical signal from the APD is usually very weak and requires multi-stage amplification and processing. The primary demodulator is another analog multiplier that performs the cross-correlation between the detected signal and a reference signal. Without the Laplace filters F1 and F2, the system is reduced to a typical spread spectrum time-resolved optical measurement system. In that case, the reference signal is simply the same PRBS (for primary modulation) delayed by a variable delay line, which is a GigaBaudics PDDL5-20. The output of the primary demodulator contains a 20 kHz signal, whose amplitude is proportional to the diffusive photon intensity at a specific time delay. This 20 kHz signal is further enhanced by a bandpass (centered at 20 kHz) amplifier and digitized by the NI DAQ device. Its amplitude is retrieved on a PC which employs a Labview program for data acquisition and secondary demodulation. The advantage of introducing the secondary modulation and demodulation is multifold. First of all, amplification stages for the 20 kHz signal can be ac coupled and the dc offset problem associated with most op-amps is eliminated. Second, the strong pink noises of semiconductor devices can be suppressed. By varying the delay at a fixed increment (e.g., 400 ps), a full time spectrum of the diffusive photons can be obtained. For each individual time delay, the typical measurement time is 2.5 ms and it takes 75 ms to acquire a TPSF with 30 time delay points.

**Figure 2:**
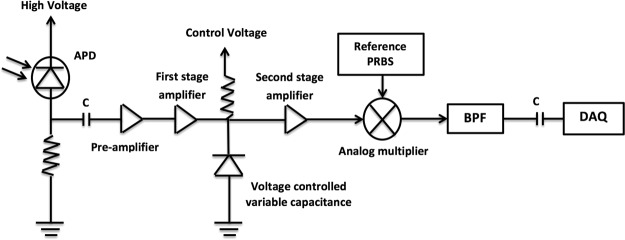
Signal path for obtaining a Laplace transformed intensity of diffusive photons. A Laplace filter is inserted between the APD preamplifier and the primary demodulator (analog multiplier).

To directly measure the Laplace transformed intensity of diffusive photons, two Laplace filters are employed to shape the reference signal or the photoelectrical signal after preamplification. Figure 2 shows the detection signal path for Laplace domain measurement when the Laplace filter F1 is enabled and F2 is disabled. F1 consists of a first-order low-pass filter, sandwiched between two RF amplifiers for impedance matching. In the varactor implementation, a voltage-controlled capacitor or varactor is used to adjust the time constant. A varactor is a diode which changes its capacitance with a varying reverse voltage across it. A digital control voltage is generated using the DAQ device to vary the capacitance from 10pF–60pF when the control voltage is changed from 10 V to 1 V. According to the theory explained in the next section, the obtained diffusive photon intensity (after first and secondary demodulation) is the Laplace transform of the TPSF at a specific Laplace frequency given by

1 $$s=1/RC,$$

where C is the varactor capacitance and *R* is 25 Ω. With the selected varactor, the Laplace parameter s ranges from 0.6 GHz to 4 GHz. To obtain smaller values of *s* fixed capacitors of values 50 pF, 90 pF, 100 pF and 200p F are used, leading to a Laplace frequency range of 0.2 GHz to 0.8 GHz.When negative s values are needed, filter F1 is disabled and F2 is enabled. In both cases, the delay line provides a constant time delay and no time-domain scanning is needed.

### Theory

The spread spectrum optical measurement method based uses cross-correlation between the sample and reference arm signal to retrieve the TPSFs. In the conventional spread spectrum method based system, the retrieved time-domain TPSF is given by:

$${I}_{TD}(\tau )=h(\tau )\ast G(\tau ),$$

where, *G*(τ) is the autocorrelation function of the PRBS, *h*(τ) is the original TPSF as a function of the delay time τ, and * denotes convolution.

In case that F2 is inserted in the reference arm and F1 is disabled (time constant set to almost zero), the PRBS signal p(t) after passing through the first order filter becomes ${p}_{1}(s,t)=p(t)\ast T(s,t)$, where $T(s,t)=s{e}^{-st}$ is the impulse response of the first order low pass filter with a time constant of 1/s. When this filtered PRBS sequence is used as the reference signal in the demodulation process, the resultant intensity can be written as

2 $$\begin{array}{ccc}{I}_{LD}(s,\tau ) & = & s{e}^{s\tau }{\int }_{0}^{\infty }(h(t)\,\ast \,G(t)){e}^{-st}\,dt\\  & = & s{e}^{s\tau }\,L\{{I}_{TD}(t)\}(s)\end{array}$$

where the operator *L*{}(*s*) defines the Laplace transform with a positive parameter s (as the time constant is always positive). When the first order low-pass filter F1 with a time constant of 1/s is enabled to shape the photoelectric signal and F2 is disabled, the demodulated intensity can be derived as

3 $$\begin{array}{ccc}{I}_{LD}(-s,\tau ) & = & s{e}^{-s\tau }{\int }_{0}^{\infty }(h(t)\,\ast \,G(t)){e}^{st}\,dt\\  & = & s{e}^{-s\tau }\,L\{{I}_{TD}(t)\}(\,-\,s),\end{array}$$

which involves the Laplace transform with a negative frequency −*s*. In both cases, the time delay remains a constant that leads to a multiplicative factor for the measured Laplace domain data. In order to obtain the Laplace transformed intensities at a few Laplace frequencies, it is no longer needed to acquire the full time spectrum across a large time delay range. Instead, the filters F1 and F2 need to properly set to repeat the direct Laplace domain measurements for a few times. The full derivation of Equations 2 and 3 is provided in Appendix 1.

### Tissue Phantom

To characterize the performance of the system, a homogeneous tissue phantom was made by diluting 20% Intralipid in a water tank. The diluted solution was 1% in concentration. The true value of reduced scattering coefficient (*μ*_*s*_′) at this concentration was around 8.9 cm^−1^. A Mie calculator was used to estimate the theoretical value of the reduced scattering coefficient of the Intralipid solution. Ink solution was added into the diluted Intralipid solution to control its absorption coefficient (*μ*_*a*_). A spectrometer (USB4000-VIS-NIR, Ocean Optics) was used to quantify the true value of the absorption coefficient. For homogeneous phantom experiments, an ink concentration of 0.01 ml/l was used. The value of the absorption coefficient for this concentration was 0.08 cm^−1^.

A cylindrical bead of diameter 0.5 cm and length 0.5 cm was embedded in the tissue phantom as an absorbing inhomogeneity. Two separate sets of experiments were carried out. In the first set we used a high absorption coefficient (1.64 cm^−1^) bead and in the second set, we used a bead with a low absorption coefficient (0.64 cm^−1^). The reduced scattering coefficient for both beads was 8.9 cm^−1^.

### COMSOL Simulation

A COMSOL based simulation was carried out to demonstrate the effect of the Laplace transform with negative Laplace frequencies on the late-arriving photons. The COMSOL model^3^ was used to predict the time-domain signal in the presence of the inhomogeneity and without inhomogeneity. For the simulation, the distance between source and detector was kept at 2.5 cm. The reduced scattering coefficient was 8.9 cm^−1^ and an absorption coefficient of 0.08 cm^−1^ was used. A cylindrical bead of diameter 0.5 cm and length 0.5 cm was used as the inhomogeneity. A MATLAB script was used to compute the Laplace transform and the perturbation was calculated using following formula,

4 $$Perturbation\,(depth,s)=100\times \frac{L(TPS{F}_{inhomo}(depth,time\,of\,flight))-L(TPS{F}_{homo}({t}ime\,of\,flight))}{L(TPS{F}_{homo}(time\,of\,flight))}$$

## Results and Discussions

In a conventional time-domain system, the complete temporal profile from the time-domain system and subsequent numerical computation of the Laplace transform are involved to obtain the Laplace transformed data. With the proposed Laplace-domain system, the direct measurement only needs to be repeated for a few Laplace frequencies. Consequently, the proposed method can significantly enhance the image acquisition speed, as it does not require the acquisition of complete temporal profiles. In the previous work, we have demonstrated that the spread spectrum time-domain system provides similar information as the conventional time-domain diffuse optical measurement system^3^. In this study, we further compare the performance of the proposed Laplace-domain system with the spread spectrum time-domain system (with both F1 and F2 disabled).

**Figure 3:**
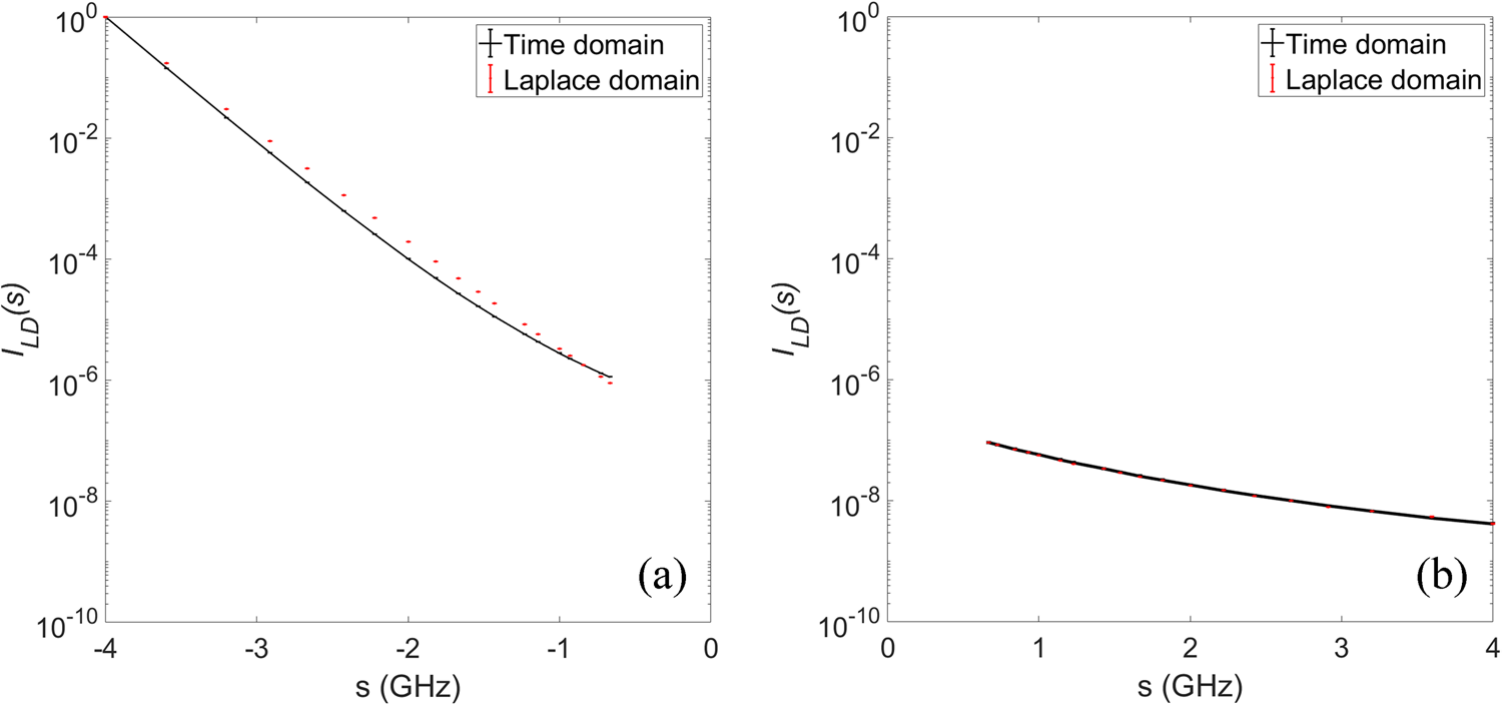
Comparison between Laplace transform data obtained from TPSFs vs Experimental Laplace-domain data (**a**) Laplace filter F1 enabled (negative **s**) (**b**) Laplace filter F2 enabled (positive **s**).

In Fig. 3, we compare the Laplace transform data obtained from the time-domain system with those from the Laplace-domain system. The measurements were carried out with the homogeneous tissue phantom. We repeated the measurement for 20 times. For the TD system, the Laplace transforms were calculated numerically from the time-domain data. Figure 3(a) shows the Laplace intensities at negative Laplace frequencies, which were obtained with the time-domain (solid line) and Laplace-domain systems (dots), respectively, with the Laplace parameters obtained with the proposed system when the first order filter is in the source arm (negative ***s*** values). In Fig. 3(b), the comparison is made for positive Laplace frequencies. All the Laplace intensities have been normalized to the intensity at the Laplace parameter of s = −4 GHz. From Fig. 3, we see a good match between the Laplace transform data obtained with the time-domain system and the proposed Laplace-domain system. To characterize the system noise, we calculated the mean and standard deviation from 20 repeated measurements. The maximum standard deviation in the Laplace transform data at each Laplace frequency was less than 1% of the mean value for both the time-domain and the proposed Laplace-domain system. It took 2.5 ms to obtain a time-domain intensity at a specific time delay. With the time-domain system, It needed 75 ms to acquire a TPSF with 30 delay time points, with which the Laplace transform can be performed numerically. To obtain a Laplace-domain intensity at a single Laplace frequency directly with the Laplace-domain system, however, needed only 2.5 ms to complete. It is therefore evident that the proposed method can afford further improved imaging speed in comparison with previous time-domain implementations without compromise the signal to noise ratio.

**Figure 4:**
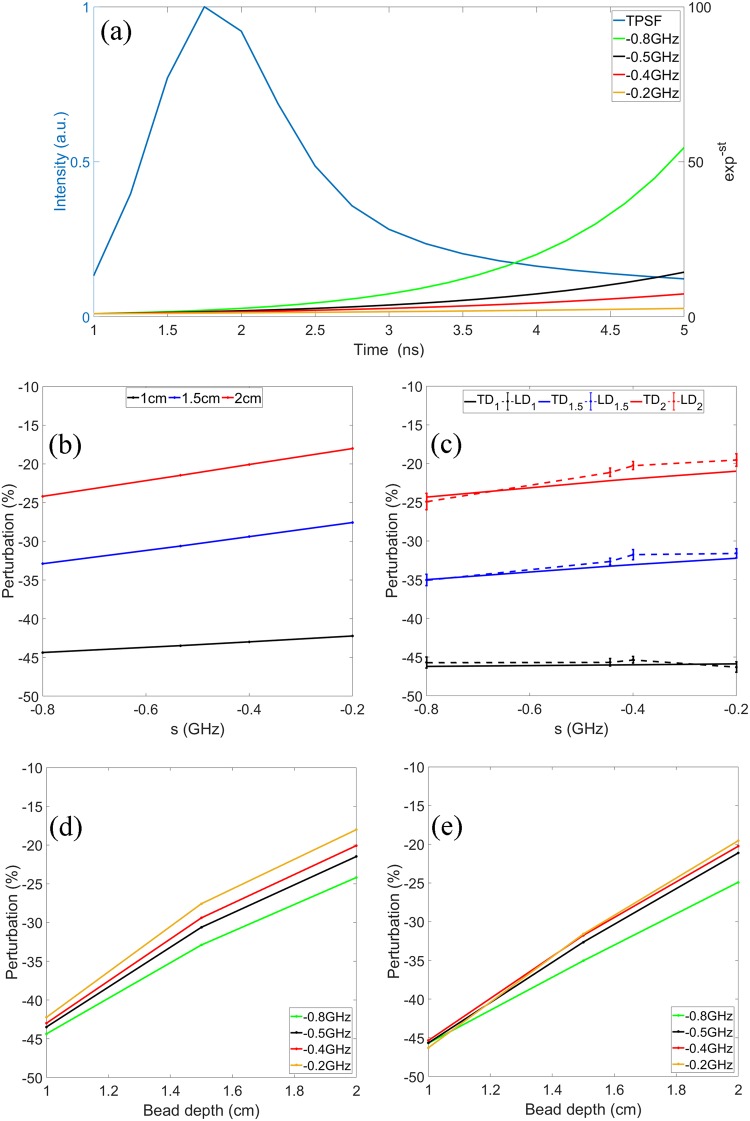
(**a**) A typical TPSF (normalized) and Laplace transform kernels of different frequencies. (**b**) Simulated perturbation at varying *s* values. (**c**) Experimental perturbations obtained from the time-domain (solid) and direct Laplace-domain (dashed) data at varying *s* values. (**d**) Simulated perturbations at varying bead depths. (**e**) Experimental perturbation obtained from the time-domain and direct Laplace-domain data at varying bead depths. The legend TD_xx_ refers to data obtained using the Laplace transform of the time-domain data for a perturbation located at the depth xx in cm. Legend LD_xx_ refers to data obtained using the proposed Laplace-domain system for a perturbation located at the depth xx in cm.

In Fig. 4(a) we plot a normalized TPSF obtained using COMSOL simulation and exponential kernels for several negative Laplace frequencies. It is clear that the negative frequencies kernels provide a higher weight to late-arriving photons when it is multiplied with the TPSFs. Therefore, the Laplace-domain intensity at a negative Laplace frequency emphasizes to the contribution of late-arriving photons. Previously, it has been demonstrated that late-arriving photons provide enhanced sensitivity to deeper perturbations^3^. As a result, Laplace transformed data with negative Laplace frequencies can provide an enhanced contrast to the inhomogeneity located in a deeper region.

To demonstrate the enhanced sensitivity due to the Laplace transform with negative Laplace frequencies, simulated and experimental results are compared in Fig 4(c) through 4(f). In both COMSOL simulation and phantom experiments, a high absorption coefficient bead (0.5 cm in diameter, 0.5 cm in length, and *μ*_a_ = 1.64 cm^−1^) was embedded in a homogeneous turbid medium with a depth from 1 to 2 cm. The optical properties of the homogeneous medium have been described in the Method section. Shown in Fig. 4(b) are simulated perturbations as functions of the Laplace frequency. For a small bead depth of 1 cm, the perturbation change is marginal within the frequency range of −0.8 to −0.2 GHz. When the bead depth increases to 1.5 and 2 cm, however, the perturbation magnitudes become signficantly higher at −0.8 GHz compared to those at −0.2 GHz. The phantom experiment results show the same trend. Plotted in Fig. 4(c) are also perturbations against the Laplace frequency, but the data were obtained experimentally from the time-domain (solid line) and Laplace-domain (dashed line) systems. The error bars indicate the standard deviations estimated from 20 repeated measurements. The simulated and experimental results from the Laplace-domain system are reorganized and plotted as functions of the bead depth in Fig. 4(e,f). All the figures suggest that measurement at lower (more negative) Laplace frequency can lead to a better sensitivity in probing the inhomogeneity in a deep region.

**Figure 5:**
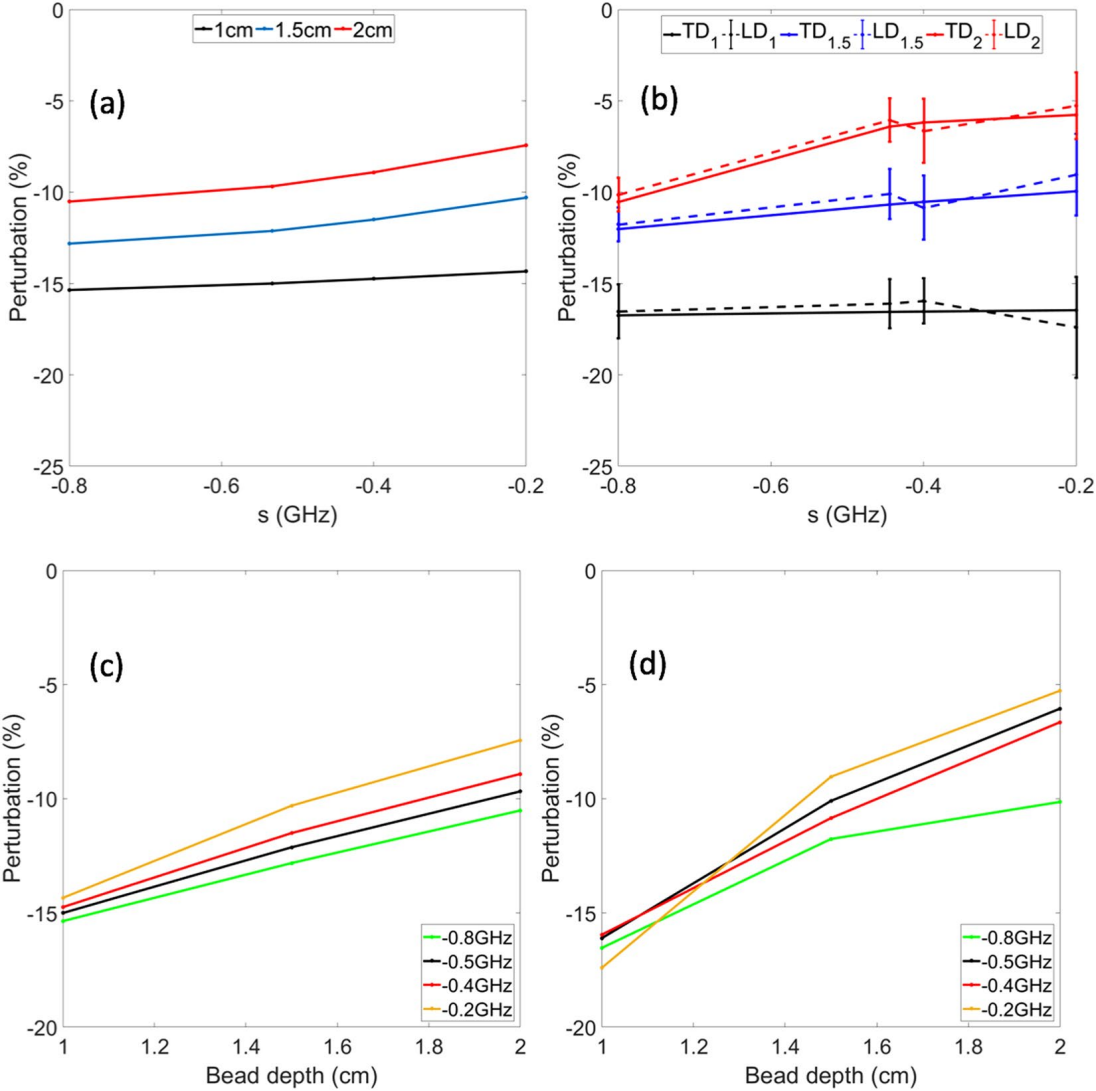
Perturbation comparison between Laplace transform data obtained with TPSFs vs. Direct Laplace transform data (Lower contrast case) (**a**) Simulated perturbation at varying *s* values. (**b**) Experimental perturbation computed from time-domain and direct Laplace-domain data at varying *s* values. (**c**) Simulated perturbation at varying bead depths. (**d**) Experimental perturbation computed from time-domain and direct Laplace-domain data at varying bead depths. The legend TD_xx_ refers to data obtained using the Laplace transform of the time-domain data for a perturbation located at the depth xx in cm. The legend LD_xx_ refers to data obtained using the proposed Laplace-domain system for a perturbation located at the depth xx in cm.

In the second set of simulation and phantom experiment studies, we used an inhomogeneity of lower contrast. In this case, an absorptive cylindrical bead (diameter: 0.5 cm, length: 0.5 cm) with an absorption coefficient of 0.64 cm^−1^ was used while the homogeneous medium remained the same. Plotted in Fig. 5(a,c) are simulated results obtained using COMSOL. Plotted in Fig. 5(b,d) are the experimental results. The experiments were repeated 20 times in order to estimate the noise levels, which are indicated by the error bars. Even with a low contrast inhomogeneity, we can see that there is a good agreement between the simulation and experimental results. The results obtained using the time-domain system and proposed Laplace-domain system also match well.

To obtain the Laplace transformed intensities from the spread spectrum method based time-domain system, we acquired TPSF with 30 data points in it. The acquisition time for a single TPSF point is 2.5 ms, thus total acquisition time for a TPSF is 75 ms. In the Laplace-domain system, we do not need to acquire a complete TPSF and then calculate the Laplace transform numerically. We can directly acquire a Laplace domain data with the acquisition time of 2.5 ms.

We believe this system can potentially provide fast and dynamic information regarding perturbations located at deep regions with high sensitivity. This system can, therefore, be potentially useful in monitoring the hemodynamics in the brain, where the perturbations are located at deep regions from the surface.

## Conclusion

In this work, we have proposed a novel Laplace-domain diffuse optical measurement system. The proposed system provides Laplace transform data without the need of obtaining a complete time-domain TPSF and therefore leading to significant increase in the data acquisition speed. We have validated the system performance by comparing the Laplace transform data obtained using the time-domain system and the proposed setup. Furthermore, we have demonstrated enhancements in the perturbation by choosing more negative Laplace frequencies.

## Electronic supplementary material

Supplementary Information
