# Supplementary material for: Laplace-domain diffuse optical measurement
Source: Sci Rep. 2018 Aug 14;8:12134. doi: 10.1038/s41598-018-30353-5 (PMC6092403; doi:10.1038/s41598-018-30353-5)
Supplement: Supplementary file 1 — Supplementary Information [file 41598_2018_30353_MOESM1_ESM.docx]

**Laplace-domain diffuse optical measurement**

**Ali Hasnain^1,*^, Kalpesh Mehta^1,*^, Xiaowei Zhou^1^, Hongsheng Li^2^,Nanguang Chen^1,^^**

^1^Department of Biomedical Engineering, National University of Singapore, Singapore 117576

**^2^**Department of Radiology, Cancer Center, Guangzhou Medical University, Guangzhou, China

[^biecng@nus.edu.sg](mailto:%5ebiecng@nus.edu.sg%20)

^*^Equal contribution

**Supplementary Information**

**Appendix 1:**

**Derivation of Laplace domain system equations:**

In the appendix first we have explain the derivation of the TPSF response for spread spectrum method based time domain system.

Lets denote $h(t)$ is the TPSF of the tissue, and the $p(t)$ is the PRBS

Now when, we excite the tissue with $p(t)$, the response from the tissue in time domain can be given by:

$R\left( t \right)=h\left( t \right)*p(t)$ (1)

A delayed PRBS signal$p(t-\tau)$ is used to demodulate the PRBS modulated response signal $R\left( t \right)$. The cross-correlation between the delayed PRBS signal and response signal provides demodulated signal:

$I_{TD}\left( \tau\right)=R(\tau)\bigotimes p(\tau)$ (2)

By inserting the Eq. (1) into the Eq. (2) and by changing the cross-correlation function to convolution function, we get following representation:

$I_{TD}\left( \tau\right)=h\left( \tau\right)*p(\tau)*p(-\tau)$ (3)

$I_{TD}\left( \tau\right)=h\left( \tau\right)*G(\tau)$ (4)

where, $G\left( \tau\right)$ is the autocorrelation of the PRBS. As the autocorrelation function of the PRBS has narrow width, we can obtain time gated TPSF details.

In the Laplace domain system, we have first order filter in either source path or the reference path. Depending upon in which arm we insert the filter, system will provide response equivalent of Laplace transform with either positive frequencies or negative frequencies.

For the case, when the first order low-pass filter is inserted in the reference arm, the PRBS signal $p\left( t \right)$ after passing through the first order filter is given by:

$$p_{1}\left( s,t \right)=p\left( t \right)*T(s,t)$$

Where, $T\left( s,t \right)={se}^{-st}$ , is the transfer function of the first order low pass filter with time constant of $1/s$, here $s$ is the Laplace parameter and its value depends of the capacitance value used in the first order filter. When, this modified PRBS sequence is used in the correlation function, the retrieved TPSF can be written as:

When we use this modified PRBS to correlate the response, we will have:

$$I_{LD}\left( s,\tau\right)=R(\tau)\bigotimes p_{1}\left( s,\tau\right)$$

$$I_{LD}\left( s,\tau\right)=R\left( \tau\right)*p_{1}\left( s,-\tau\right)$$

$$I_{LD}\left( s,\tau\right)=h\left( \tau\right)*p\left( \tau\right)*p\left( -\tau\right)*T(s,-\tau)$$

$$I_{LD}\left( s,\tau\right)=h\left( \tau\right)*G(\tau)*T(s,-\tau)$$

Using the relationship, $A*B(t)=\int_{0}^{\infty} A\left( \beta\right).B(t-\beta)d\beta$

$$I_{LD}\left( s,\tau\right)=\int_{0}^{\infty} (h\left( t \right)*G(t))T(s,t-\tau)dt$$

$$I_{LD}\left( s,\tau\right)=s\int_{0}^{\infty} (h\left( t \right)*G(t))e^{-s(t-\tau)}dt$$

$$I_{LD}\left( s,\tau\right)=se^{s\tau}\int_{0}^{\infty} (h\left( t \right)*G(t))e^{-st}\mathrm{dt}$$

$$I_{LD}\left( s,\tau\right)=se^{s\tau}L\left\{ I_{TD}\left( t \right) \right\}(s)$$

Where, the $L\left\{ I_{TD}\left( t \right) \right\}(s)$is the Laplace transform of the time domain TPSF with a positive parameter *s*.

In the case, when the first order low-pass filter is inserted in the source arm the measured TPSF can be derived as:

In this case, the response signal detected by the APD can be written as:

$$R\left( s,t \right)=h\left( t \right)*p\left( t \right)*T(s,t)$$

here, $p\left( t \right)*T(s,t)$ is the excitation signal for the sample. The demodulated signal is:

$$I_{LD}\left( -s,\tau\right)=R(s,\tau)\bigotimes p\left( \tau\right)$$

$$I_{LD}\left( -s,\tau\right)=R\left( s,\tau\right)*p\left( -\tau\right)$$

$$I_{LD}\left( -s,\tau\right)=h\left( \tau\right)*p\left( \tau\right)*T\left( s,\tau\right)*p\left( -\tau\right)$$

$$I_{LD}\left( -s,\tau\right)=h\left( \tau\right)*G(\tau)*T(s,\tau)$$

Using the relationship, $A*B(t)=\int_{0}^{\infty} A\left( \beta\right).B(t-\beta)d\beta$

$$I_{LD}\left( -s,\tau\right)=\int_{0}^{\infty} \left( h\left( t \right)*G\left( t \right) \right)T(s,\tau-t)dt$$

$$I_{LD}\left( -s,\tau\right)=s\int_{0}^{\infty} (h\left( t \right)*G(t))e^{-s(\tau-t)}dt$$

$$I_{LD}\left( -s,\tau\right)=se^{-s\tau}\int_{0}^{\infty} (h\left( t \right)*G(t))e^{st}dt$$

$$I_{LD}\left( -s,\tau\right)=se^{-s\tau}\int_{0}^{\infty} (h\left( t \right)*G(t))e^{-(-s)t}dt$$

$$I_{LD}\left( -s,\tau\right)=se^{-s\tau}L\left\{ I_{TD}\left( t \right) \right\}\left( -s \right)$$

Where, the $L\left\{ I_{TD}\left( t \right) \right\}(-s)$is the Laplace transform of the time domain TPSF with a negative parameter -*s*.
